# Rapid Accumulation of Virulent Rift Valley Fever Virus in Mice from an Attenuated Virus Carrying a Single Nucleotide Substitution in the M RNA

**DOI:** 10.1371/journal.pone.0009986

**Published:** 2010-04-01

**Authors:** John C. Morrill, Tetsuro Ikegami, Naoko Yoshikawa-Iwata, Nandadeva Lokugamage, Sungyong Won, Kaori Terasaki, Aya Zamoto-Niikura, C. J. Peters, Shinji Makino

**Affiliations:** 1 Department of Microbiology and Immunology, The University of Texas Medical Branch at Galveston, Galveston, Texas, United States of America; 2 Department of Pathology, The University of Texas Medical Branch at Galveston, Galveston, Texas, United States of America; University of Hong Kong, Hong Kong

## Abstract

**Background:**

Rift Valley fever virus (RVFV), a member of the genus *Phlebovirus* within the family *Bunyaviridae*, is a negative-stranded RNA virus with a tripartite genome. RVFV is transmitted by mosquitoes and causes fever and severe hemorrhagic illness among humans, while in livestock it causes fever and high abortion rates.

**Methodology/Principal Findings:**

Sequence analysis showed that a wild-type RVFV ZH501 preparation consisted of two major viral subpopulations, with a single nucleotide heterogeneity at nucleotide 847 of M segment (M847); one had a G residue at M847 encoding glycine in a major viral envelope Gn protein, while the other carried A residue encoding glutamic acid at the corresponding site. Two ZH501-derived viruses, rZH501-M847-G and rZH501-M847-A, carried identical genomic sequences, except that the former and the latter had G and A, respectively, at M847 were recovered by using a reverse genetics system. Intraperitoneal inoculation of rZH501-M847-A into mice caused a rapid and efficient viral accumulation in the sera, livers, spleens, kidneys and brains, and killed most of the mice within 8 days, whereas rZH501-M847-G caused low viremia titers, did not replicate as efficiently as did rZH501-M847-A in these organs, and had attenuated virulence to mice. Remarkably, as early as 2 days postinfection with rZH501-M847-G, the viruses carrying A at M847 emerged and became the major virus population thereafter, while replicating viruses retained the input A residue at M847 in rZH501-M847-A-infected mice.

**Conclusions/Significance:**

These data demonstrated that the single nucleotide substitution in the Gn protein substantially affected the RVFV mouse virulence and that a virus population carrying the virulent viral genotype quickly emerged and became the major viral population within a few days in mice that were inoculated with the attenuated virus.

## Introduction

Rift Valley fever virus (RVFV), a member of the genus *Phlebovirus* within the family *Bunyaviridae*, causes periodic outbreaks among livestock and humans in sub-Saharan African countries [1]. RVFV infection results in high mortality and abortion rates in domestic ruminants with severe hepatic diseases. It also causes an acute febrile myalgic syndrome, a hemorrhagic syndrome, ocular disease, and encephalitis in humans [2], [3]. Transmission of RVFV to humans is primarily mosquito-borne or due to direct contact with infected animal blood, tissues or products of abortion. Since the late 1970s, several major outbreaks of Rift Valley fever have occurred outside of sub-Saharan Africa, e.g., in Egypt [4], Madagascar [5], Saudi Arabia, and Yemen [2], [6]. The most recent outbreak was reported in Kenya and resulted in a high reported case-fatality ratio in infected humans [7].

RVFV has a single-stranded, tripartite RNA genome composed of the L, M, and S segments. The L segment is of negative polarity encoding the RNA-dependent RNA polymerase (L). The anti-viral-sense M segment encodes two envelope glycoproteins, Gn and Gc, and two accessory proteins,14-kDa NSm that suppresses virus-induced apoptosis [8] and the 78-kDa protein. The S segment uses an ambisense strategy for gene expression; a nonstructural protein, NSs, is translated from viral-sense mRNA, while N protein is expressed from anti-viral-sense mRNA [1]. N protein and L protein are essential for viral RNA synthesis [1] and NSs protein suppresses host innate immune functions by suppressing host gene expression [9], including interferon-β [10], and promoting PKR degradation [11], [12].

Experiments using reassortant viruses between an attenuated MP-12 strain of RVFV and wild-type (wt) RVFV suggested that RVFV virulence characteristics in the mouse are under polygenic control [13]. Further studies using reassortant viruses between the wt ZH548 strain of RVFV and an attenuated RVFV isolate clone 13, a plaque clone variant of the wt 74HB59 strain carrying a deletion of 69% of the NSs gene [14], suggested that RVFV mouse virulence is controlled by the S segment [15]. The importance of NSs on viral virulence was confirmed by using a wt RVFV lacking the entire NSs gene [16], [17]. Studies using the wt RVFV lacking the NSm gene showed that NSm also affected virus virulence in rats [18]. In contrast, how Gn and Gc envelope proteins and L protein contribute to viral virulence is unknown.

The present study revealed that wt RVFV ZH501 stock, which was originally isolated from a patient during the 1977 outbreak of RVFV in Egypt, was made up of two major virus populations with single nucleotide substitution within the Gn gene and that a single nucleotide difference in the Gn gene of wt RVFV substantially affected viral mouse virulence. Furthermore, we observed a remarkably rapid emergence and accumulation of the virulent-type virus in the mice that had been inoculated with a low virulent-type RVFV.

## Results

### Presence of two major viral subpopulations in wt ZH501 virus stock

**Figure 1:**
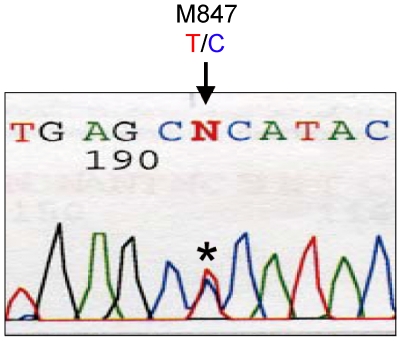
Sequence analysis of the RT-PCR product of intracellular viral-specific M RNA. The sequence of the viral-sense M segment of ZH501 shows sequence heterogeneity, T and C, at M847 (asterisk).

Sequence analysis of the RT-PCR products of intracellular ZH501-specific RNAs showed sequence homogeneity in all three RNA segments, except for nucleotide 847 of the anti-viral sense M segment RNA (M847), which consisted of a mixture of A and G residues (Fig. 1). Of 35 independent, cloned RT-PCR products of a region including M847, 18 clones and 17 clones had A and G, respectively, at M847, which suggested to us that the ZH501 virus stock consisted of roughly a 1-to-1 mixture of two major viral subpopulations, one carrying A and the other carrying G at M847. The virus carrying A residue at M847 encodes glutamic acid at amino acid 277 (Glu277) of the Gn envelope protein, while that carrying the G residue encodes glycine (Gly277). Sequence analysis of the cloned PCR products also showed other sequence heterogeneities, including silent mutations and those that altered amino acid sequences, within the M segment (data not shown), revealing a quasispecies nature of the ZH501 virus stock. None of these other nucleotide heterogeneities were shared among clones (data not shown).

### Recovery of rZH501-M847-A and rZH501-M847-G from cDNAs

To know the effects of the nucleotide difference at M847 on biological properties of the virus, we have recovered ZH501 carrying A at M847 (rZH501-M847-A) and that carrying G (rZH501-M847-G) at the corresponding site using a reverse genetics system of ZH501, in which endogenously expressed T7 polymerease in BHK/T7-9 cells drives expression of viral RNAs and proteins from the transfected plasmids. Sequence analysis of the entire L, M and S RNA segments of rZH501-M847-A and rZH501-M847-G revealed that both viruses had the expected primary sequences.

**Figure 2:**
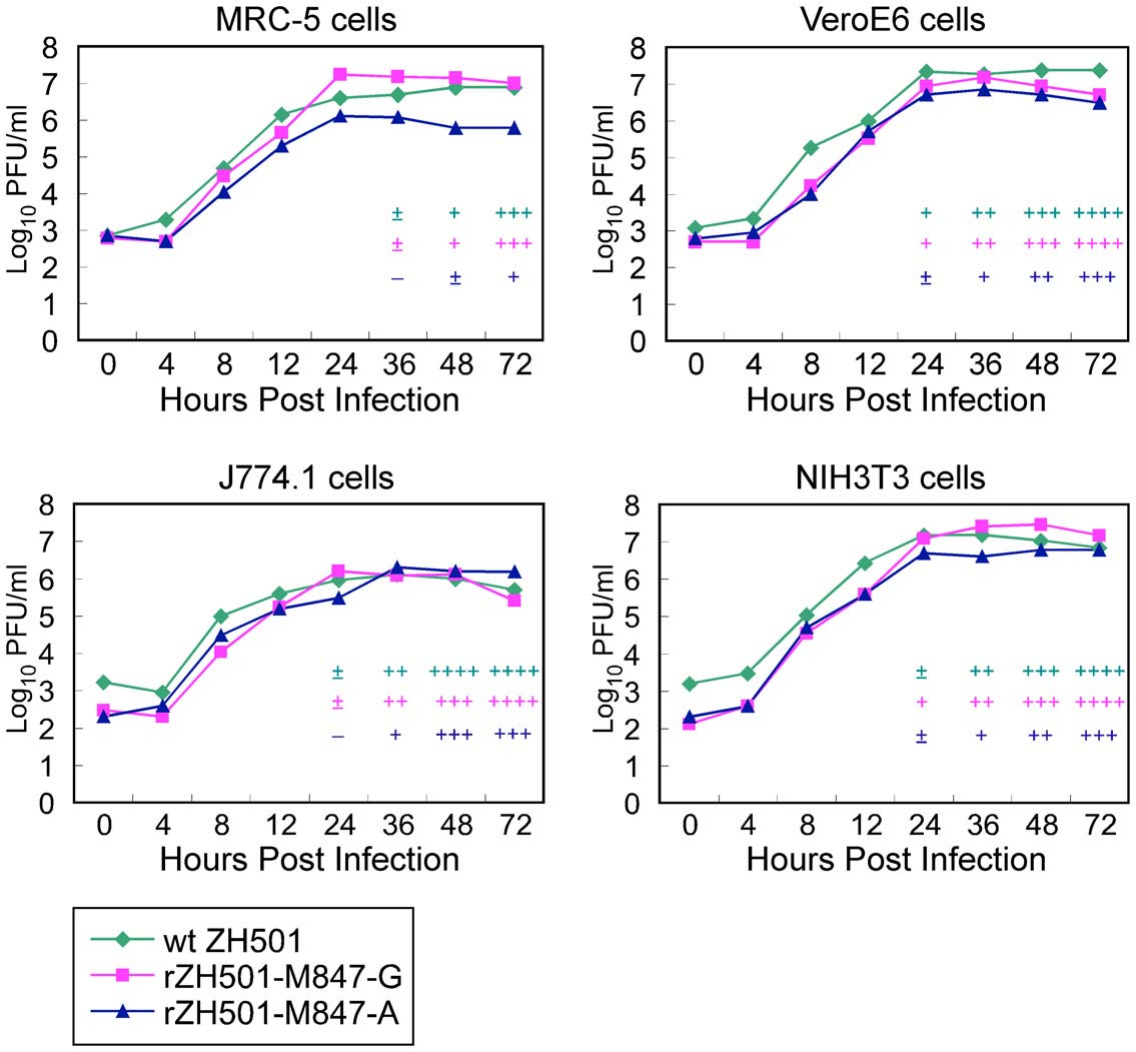
Growth curve of ZH501, rZH501-847-A and rZH501-847-A in various cell lines. VeroE6 cells, NIH3T3 cells, J774.1 cells and MRC-5 cells were inoculated with ZH501, rZH501-847-A or rZH501-847-A at an moi of 0.02. Culture fluids were collected and virus titers were determined by a plaque assay using VeroE6 cells. Virus growth curve of one representative experiment for each cell type is shown. Progression of CPE is represented by the symbols: +, ++, +++ or +++. +  =  up to 25% of cell sheet affected showing cytolysis and swollen cells. ++  =  up to 50% of cell sheet affected showing lysed cells, floating cells and cell debris +++  =  up to 75% of cell sheet affected showing significant cell lysis and increased amounts of cell debris in culture fluid. ++++  =  cell sheet destroyed with complete cytolysis.

No substantial differences in the plaque sizes and morphologies were detected among ZH501, rZH501-M847-G and rZH501-M847-A in both Vero cells and MRC-5 cells (data not shown). ZH501, rZH501-M847-G and rZH501-M847-A showed similar replication kinetics in VeroE6 cells, mouse 3T3 cells and mouse macrophage-derived J774.1 cells (Fig. 2), while titers of rZH501-M847-G were roughly 10 times higher than those of rZH501-M847-A in human lung fibroblast MRC-5 cells from 24 h to 72 h p.i. (Fig. 2). Also there was a trend that CPE induced by rZH501-M847-A was less prominent CPE than that induced by rZH501-M847-G or ZH501 at a given time in all four cell lines (Fig. 2). Unpaired-t-tests of three independent experiments examining the replication kinetics of rZH501-M847-G and rZH501-M847-A in MRC-5 cells from 0 h to 72 h p.i. revealed that the titer of rZH501-M847-G was a significantly higher (P<0.01) than that of rZH501-M847-A at 72 h p.i. (Fig. S1).

### Mouse virulence of ZH501, rZH501-M847-A and rZH501-M847-G

**Figure 3:**
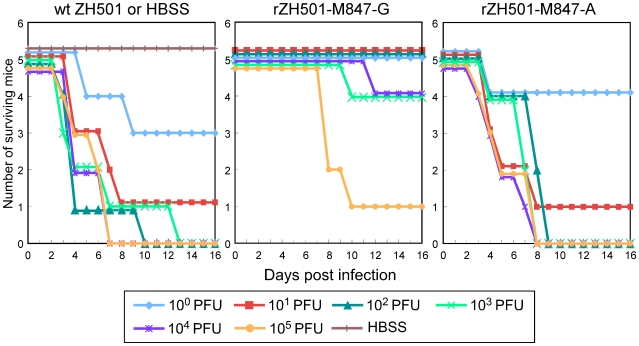
The virulence of ZH501, rZH501-M847-A and rZH501-M847-G in mice. Five-week-old female CD-1 mice were inoculated i.p. with 10^0^, 10^1^, 10^2^, 10^3^, 10^4^ or 10^5^ PFU of ZH501, rZH501-M847-A or rZH501-M847-G. Control mice were inoculated with HBSS. Mortality was recorded daily for 28 days p.i. No mice died after 14 days p.i.

We examined the virulence of rZH501-M847-G, rZH501-M847-A and ZH501 by intraperitoneal (i.p.) inoculation of 10^0^, 10^1^, 10^2^, 10^3^, 10^4^ or 10^5^ plaque-forming units (PFU) of each virus into five 5-week-old female CD-1 mice. Hanks' balanced salt solution (HBSS) was inoculated into control mice. These mice were observed daily for 28 days p.i. (Fig. 3). None of the HBSS-inoculated mice died, while inoculation of 10^2^ to 10^5^ PFU of ZH501 resulted in the death of all of the mice within 13 days p.i., and one and three mice survived after inoculation of 10^1^ and 10^0^ PFU, respectively. All mice that were inoculated with 10^2^ to 10^5^ PFU of rZH501-M847-A died within 9 days p.i., and infection with 10^1^ and 10^0^ PFU resulted in the survival of one and four mice, respectively. To our surprise, none of the rZH501-M847-G infected mice died after inoculation of 10^0^ to 10^2^ PFU, four out of five mice survived after inoculation of 10^4^ and 10^3^ PFU, and one mouse survived after infection with 10^5^ PFU. These data demonstrated that ZH501 and rZH501-M847-A were highly virulent to mice, whereas rZH501-M847-G had reduced mouse virulence.

**Figure 4:**
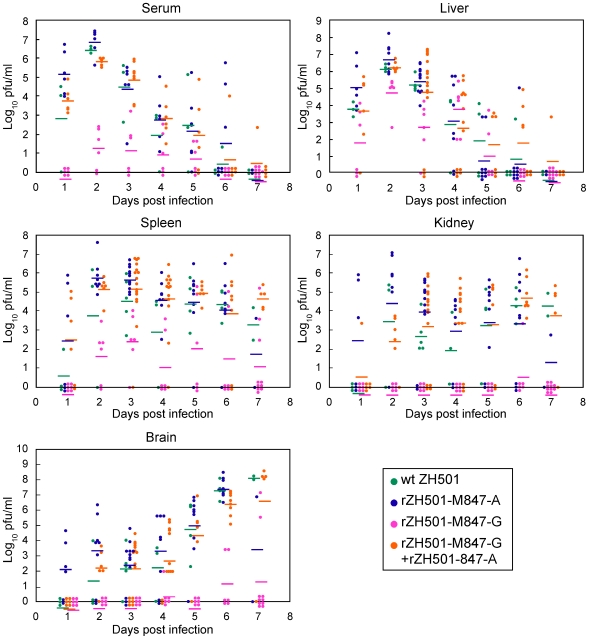
Virus titers in the various organs and serum of infected mice. Five-week-old, female CD-1 mice were inoculated i.p. with 10^5^ PFU of ZH501, rZH501-M847-A, rZH501-M847-G or a mixture of rZH501-M847-A and rZH501-M847-G. At 1 through 7 days p.i., serum, liver, spleen, kidney and brain were collected from infected mice, and virus titers were individually determined by plaque assay. Two to sixteen mice were used for each time point. In some cases, samples were collected from dead mice. They were: ZH501-infected mice–2 at day 3 p.i., 1 at 5 day p.i. and 1 at day 6 p.i.; rZH501-M847-G-infected mice–1 at day 7 p.i.; rZH501-M847-A-infected mice–2 at days 2 and 4 p.i., 5 at day 3 p.i., 3 at day 5 p.i., and 4 at day 6 p.i.; and rZH501-M847-G/rZH501-M847-A-co-infected mice–8 at day 3 p.i., 7 at day 6 p.i., and 2 at day 6 p.i.

To know the kinetics and titers of virus replication in various organs, mice were inoculated i.p. with 10^5^ PFU of rZH501-M847-G, rZH501-M847-A, ZH501 and a mixture of 10^5^ PFU of rZH501-M847-G and the same titer of rZH501-M847-A. Virus titers in livers, spleens, kidneys, sera and brains were determined from 1 to 7 days p.i.; two to sixteen mice were used for each time point (Fig. 4). The mice infected with ZH501, rZH501-M847-A, and the mixture of rZH501-M847-G and rZH501-M847A exhibited rapid, efficient virus replications in the liver early in infection, high titers of viremia from 1 to 5 days p.i. and a gradual increase of efficient virus replication in the brain, where the virus titers exceeded 10^6^ PFU/g at day 6 p.i. Efficient virus replication also occurred in the spleens and the kidneys, and all mice died by day 8 p.i. The mice infected with rZH501-M847-G also showed rapid virus replication in the liver, and yet the maximum liver virus titer was approximately 10 times lower than that in the mice infected with rZH501-M847-A, ZH501 and the mixture of rZH501-M847-G and rZH501-M847-A. Also rZH501-M847-G titers in the sera, kidneys, spleens and brains were substantially lower than the virus titers in the corresponding organs of mice that were infected with rZH501-M847-A, ZH501 and the mixture of rZH501-M847-G and rZH501-M847-A. Unpaired-t-test results showed that virus titers at days 1 to 3 in the serum and the liver, days 2 to 4 in the spleen, days 2 to 5 in the kidney and days 2 to 6 in the brain of rZH501-M847-G-infected mice were statistically lower (p<0.01) than the titers in the corresponding organs of rZH501-M847-A-infected mice.

### Neutralizing antibody titers in the infected mice

**Figure 5:**
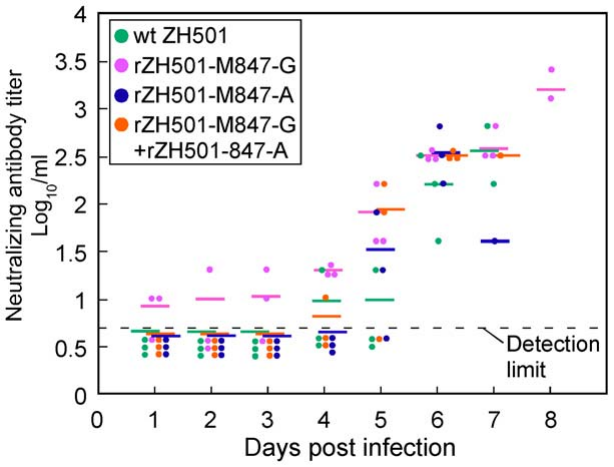
Neutralizing antibody titers in the infected mice. Serum neutralizing antibody titers of five-week-old female CD-1 mice inoculated i.p. with 10^5^ PFU of ZH501, rZH501-M847-A, rZH501-M847-G or a mixture of rZH501-M847-A and rZH501-M847-G. Dashed line denotes the minimum neutralizing antibody detection level.

We next examined serum-neutralizing antibody titers in the infected mice; ZH501 was used for the neutralizing antibody assay. For each virus group, 19 to 23 mice were used. As shown in Fig. 5, all mice that were inoculated i.p with 10^5^ PFU of rZH501-M847-A, ZH501 or a mixture of 10^5^ PFU of rZH501-M847-G and the same titer of rZH501-M847-A had less than detectable levels of neutralizing antibodies until day 3 p.i. In contrast, rZH501-M847-G-infected mice showed low, but detectable levels of neutralizing antibodies early in infection; moreover, a low titer of neutralizing antibody was detected as early as 1 day p.i. in some of rZH501-M847-G-infected mice. Although neutralizing antibody titers increased in infected mice after day 5 p.i., there was a trend that rZH501-M847-G-infected mice had higher neutralizing antibody titers than did other infected mice. No neutralizing antibodies were detected in sham-infected mice (data not shown).

### Cross-neutralization analysis of rZH501-M847-A, rZH501-M847-G and ZH501

We performed cross-neutralization assays to determine how efficiently antiserum from rZH501-M847-G-infected mice and that from rZH501-M847-A-infected mice could neutralize rZH501-M847-G, rZH501-M847-A, and ZH501. Anti-rZH501-M847-G serum demonstrating an 80% neutralizing antibody titer (PRNT_80_ titer) of 1∶640 to ZH501 and anti-rZH501-M847-A serum demonstrating a PRNT_80_ titer of 1∶80 to ZH501 were obtained from rZH501-M847-G-infected mice and rZH501-M847-A-infected mice, respectively. We used as controls, diluent, normal mouse serum and convalescent goat anti-ZH501 serum showing a PRNT_80_ titer of 1∶5,120 to ZH501. In this assay, virus titers were determined after overnight incubation of 100 µl of approximately 5.0 log_10_ PFU/ml of rZH501-M847-G, rZH501-M847-A, and ZH501 with the same volume of undiluted antiserum or diluent at 4°C (Table 1). Goat anti-ZH501 serum efficiently neutralized all viruses, while incubation of rZH501-M847-A and rZH501-M847-G with the normal mouse serum resulted in small reductions of the virus titers. Anti-rZH501-M847-G serum and anti-rZH501-M847-G serum neutralized the three viruses at different efficiencies. The differences between the virus titers (in log_10_PFU/ml) after incubation with each antiserum and those after incubation with normal serum are shown within parentheses in Table 1. The anti-rZH501-M847-G serum reduced the titer of rZH501-M847-G by 1.63 log_10_ PFU/ml, while it reduced the titer of rZH501-M847-A and ZH501 by 0.82 and 0.52 log_10_ PFU/ml, respectively. The anti-rZH501-M847-A serum reduced the titers of rZH501-M847-A, rZH501-M847-G and ZH501 by 1.60, 0.7, and 1.0 log_10_ PFU/ml, respectively. These data demonstrated that mouse antiserum neutralized the homologous virus to a similar extent, regardless of the PRNT_80_ titers, that antiserum neutralized the heterologous virus equally but to a lesser degree than the homologous virus, and that anti-rZH501-M847-A serum was able to neutralize the titer of ZH501 to a slightly greater degree than anti-rZH501-M847-G serum; however, neither serum neutralized ZH501 equal to the neutralization of their homologous viruses.

**Table 1:** Cross-neutralization assays.

Virus	Diluent	Normal mouse serum	Anti-rZH501-M847-G [1∶640]*	Anti-rZH501-M847-A [1∶80]	Goat anti-ZH501 [1∶5,120]
rZH501-M847-G	4.70**	4.48	2.85 (1.63)***	3.78 (0.70)	<0.7
rZH501-M847-A	4.57	4.30	3.48 (0.82)	2.70 (1.60)	<0.7
ZH501	4.90	5.00	4.48 (0.52)	4.00 (1.00)	<0.7

*PRNT_80_ titer to ZH501.

**Numbers represent virus titers in log_10_PFU/ml that were obtained after incubation with diluent, normal mouse serum or antisera.

***Numbers within the parenthesis represent differences between the virus titers in log_10_PFU/ml after incubation with each antiserum and those after incubation with normal mouse serum.

### Histopathological and immunohistochemical (IHC) examinations

**Figure 6:**
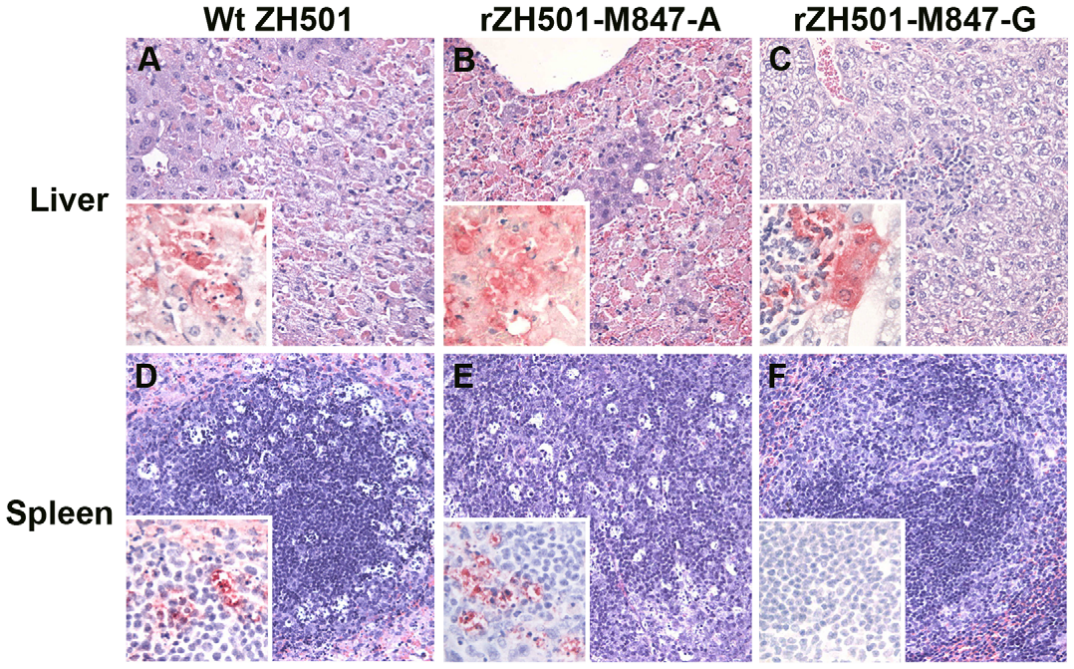
Histopathology and immunohistochemistry (IHC) of mice infected with ZH501 (A and D), rZH501-M847-A (B and E) and rZH501-M847-G (C and F). (A) Liver histopathology and IHC (inset) from mice infected with ZH501 on day 3 p.i. The majority of the hepatocytes were necrotic (hematoxylin and eosin staining) (magnification, ×200, inset: ×400). (B) Liver histopathology and IHC (inset) from mice infected with rZH501-M847-A on day 3 p.i. The samples showed severe hemorrhages in the livers due to disrupting hepatocytes (magnification, ×200, inset: ×400). (C) Liver histopathology and IHC (inset) from mice infected with rZH501-M847-G on day 3 p.i. Mice had focal necrosis in the liver (magnification, ×200, inset: ×400). (D) Spleen histopathology and IHC (inset) from mice infected with ZH501 on day 3 p.i. Depletion of lymphocytes in the white pulp was present (magnification, ×200, inset: ×400). (E) Spleen histopathology and IHC (inset) of mice infected with rZH501-M847-A. The lesion was the same as that of ZH501-infected mice (magnification, ×200, inset: ×400). (F) Spleen histopathology and IHC (inset) of mice infected with rZH501-M847-G (magnification, ×200, inset: ×400). No lesions or viral antigens were detected in the spleens of mice infected with rZH501-M847-G.

To further understand the pathogenesis of rZH501-M847-G and rZH501-M847-A, we performed histopathological and IHC analyses of various organs of mice inoculated i.p. with 10^5^ PFU of rZH501-M847-G, rZH501-M847-A, ZH501 or a mixture of 10^5^ PFU of rZH501-M847-G and the same titer of rZH501-M847-A at various times p.i. (Figs. 6 and S2).

The livers of rZH501-M847-A-infected mice showed multifocal hepatocellular necrosis with prominent aggregating mononuclear inflammatory cells and sparsely scattered viral antigens throughout the liver lobe as early as day 2 p.i. On day 3 p.i., most of the hepatocytes were necrotic and positive for viral antigens (Fig. 6B). Also, severe hemorrhages were noted throughout the lobe. After day 4 p.i., the livers from mice examined showed less severe hepatocellular damage than livers taken earlier, and the livers examined on day 6 p.i. showed near complete resolution. ZH501-infected mice presented with similar lesions and distribution of viral antigens, and yet the progress of the lesions was somewhat slower than those in rZH501-M847-A-infected mice (Fig. 6A). Although rZH501-M847-G-infected mice had focal-to-multifocal hepatocellular necrosis from days 2 to 4 p.i (Fig. 6C), they exhibited mild hepatic lesions compared to ZH501-infected mice and rZH501-M847-A-infected mice. Among mice inoculated with the mixture of rZH501-M847-G and rZH501-M847-A, one showed severe hepatic lesions like those of rZH501-M847-A-infected mice, while others had mild hepatic lesions like that of rZH501-M847-G-infected mice (data not shown).

The spleens of rZH501-M847-A-infected mice (Fig. 6E), ZH501-infected mice (Fig. 6D) and mice inoculated with the mixture of rZH501-M847-G and rZH501-M847-A (data not shown) were characterized by a depletion of lymphocytes in the white pulp from day 2 p.i. to day 5 or 6 p.i., with maximum depletion seen on day 3 p.i., and the presence of viral antigens in the white pulp from days 2 to 4 p.i. No lesions and viral antigens were detected in the spleens of rZH501-M847-G-infected mice (Fig. 6F).

All infected mice, except for rZH501-M847-G-infected mice, had moderate, non-suppurative encephalitis throughout their brains from day 5 p.i. (Fig. S2A). Viral antigens were detected as early as day 2 p.i. in the endothelial cells of some mice. After day 5 p.i., viral antigens were detected in neurons at the thalamus, and spread to neurons of the surrounding areas (Fig. S2A, inset). The lesions were diagnosed as mild meningitis or mild encephalitis in the brains of rZH501-M847-G-infected mice (Fig. S2E), and viral antigen was detected throughout the brains after day 7 p.i.

Kidneys of some of rZH501-M847-A-infected mice and the mice infected with the mixture of rZH501-M847-G and rZH501-M847-A showed pyknotic cells in glomeruli on day 3 p.i (Fig. S2B). Most of the infected mice had viral antigens in the smooth muscle of interlobular and arcuate arteries from day 2 p.i., and in the mesangium of glomeruli on days 3 and 4 p.i (Fig. S2C, D). Although no pathology was detected in the kidneys of the ZH501-infected mice, viral antigens were detected from day 3 in the mesangium of glomeruli and in the smooth muscle of interlobular and arcuate arteries from days 3 to 7 p.i. The kidneys of rZH501-M847-G-infected mice showed no pathology. Some of the infected mice exhibited viral antigens from days 5 to 7 day p.i. in the smooth muscle of interlobular and arcuate arteries (Fig. S2H), whereas no viral antigens were detected in the glomeruli (Fig. S2G).

In summary, there were rough correlations between the presence of viral antigens and viral titers in the given organs, and rZH501-M847-G-infected mice had delayed development of lesions or showed less severe lesions than did other infected mouse groups.

### Emergence and accumulation of viruses carrying A residue at M847 in rZH501-M847-G-infected mice

**Figure 7:**
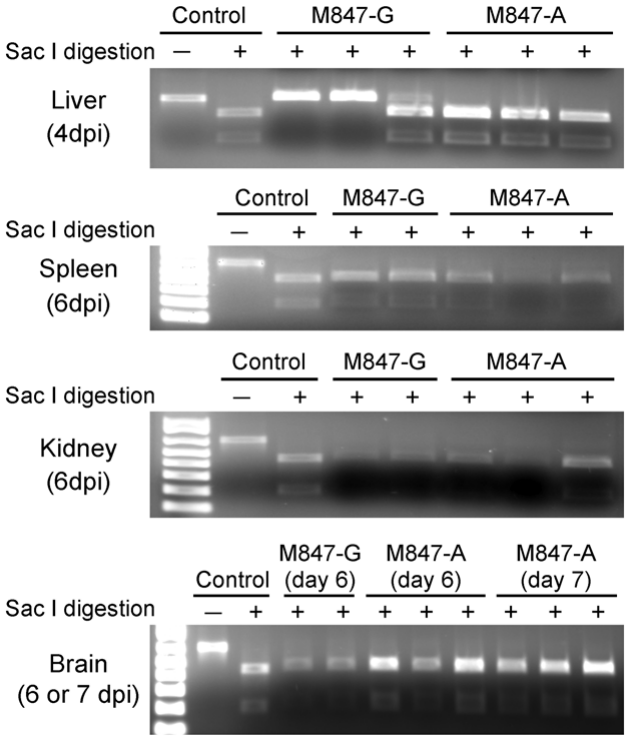
Sac I digestion of the RT-PCR products. Five-week-old female CD-1 mice were infected i.p. with 10^5^ PFU of rZH501-M847-G or rZH501-M847-A. Total RNAs from 3 livers at 4 day p.i., 3 spleens at day 6 p.i., 3 kidneys at day 6 p.i., 3 brains at day 6 p.i., and 3 brains at day 7 p.i. were collected and subjected to RT-PCR analysis. In the samples where RT-PCR products were obtained, RT-PCR products were digested with Sac I and subjected to agarose gel electrophoresis. As controls, PCR products that were obtained from plasmid encoding M segment of rZH501-M847-A were digested with Sac I or incubated in the absence of Sac I (Control).

We next examined whether the viruses that accumulated in infected mice retained the input-virus genome sequence at M847. To this end, we took advantage of the presence of a naturally occurring Sac I site (GAGCTC), which includes the A residue (underlined) at M847 of rZH501-M847-A. Due to single nucleotide substitution, the corresponding region of rZH501-M847-G lacked this restriction enzyme site. Mice were infected i.p. with 10^5^ PFU of rZH501-M847-G or rZH501-M847-A (n = 9 for each group). Organs were isolated at various times p.i., and RT-PCR products that included M847 were subjected to Sac I digestion. For each virus group, we collected 3 livers at 4 day p.i., 3 spleens, 3 kidneys and 3 brains at day 6 p.i., and 3 brains at day 7 p.i. RT-PCR products of the expected size were obtained from all samples of rZH501-M847-A-infected mice, and all were fully susceptible to Sac I digestion (Fig. 7), demonstrating that replicating rZH501-M847-A retained A residue at M847 in the infected mice. We were unable to obtain RT-PCR products from one spleen sample at day 6 p.i., one kidney sample at day 6 p.i., one brain sample at day 6 p.i., and one brain sample at day 7 p.i. from rZH501-M847-G-infected mice (data not shown), probably due to low virus titers in these samples. Two liver samples at day 4 p.i., were resistant to Sac I digestion (Fig. 7). Unexpectedly, the RT-PCR products of one liver sample were a mixture of Sac I-susceptible PCR product and Sac I-resistant products (Fig. 7) and all other RT-PCR products, including two spleen samples at day 6 p.i., two brain samples at day 6 p.i., and two brains at day 7 p.i. (data not shown) were susceptible to the Sac I digestion (Fig. 7). These data strongly suggested an accumulation of rZH501-M847-A-type virus carrying A residue at M847, in rZH501-M847-G infected mice at 4 days p.i. in livers and 6 days p.i. in spleens and brains.

**Figure 8:**
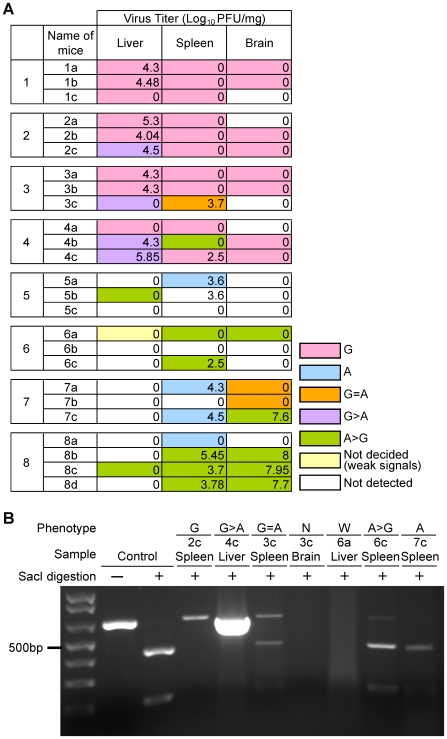
Accumulation of viruses carrying the genotype of rZH501-M847-A in rZH501-M847-G-infected mice. Five-week-old female CD-1 mice were infected i.p. with 10^5^ PFU of rZH501-M847-G and liver, spleen and brain were collected every day from day 1 to day 8 p.i. Virus titer in each sample was determined by a plaque assay. RT-PCR products were obtained from total RNAs in these samples and then subjected to Sac I digestion. (A) Color and number in each box represent the genotype of major viruses and the virus titer in Log_10_PFU/g of tissue, respectively, in each mouse. Color legend: G  =  genotype of rZH501-M847-G. A  =  genotype of rZH501-M847-A. G = A  =  a mixture of similar amounts of rZH501-M847-G genotype and rZH501-M847-A genotype. G>A  =  viruses carrying the rZH501-M847-G genotype were more abundant than those carrying the rZH501-M847-A genotype. A>G  =  viruses carrying the rZH501-M847-A genotype were more abundant than those carrying the rZH501-M847-G genotype. Not decided  =  the major genotype of viruses was not determined due to weak RT-PCR signals. Not detected  =  RT-PCR products were not obtained. (B) Agarose gel electrophoresis of the RT-PCR products that underwent SacI digestion. Only representative samples are shown. The mouse identification number in the gel is shown in (A). Control  =  PCR products that were obtained from plasmid encoding M segment of rZH501-M847-A were digested with Sac I or incubated in the absence of Sac I. N  =  Not detected. W  =  Not decided.

To learn how quickly rZH501-M847-A-type virus accumulated in various organs of rZH501-M847-G-infected mice, we inoculated 25 mice i.p. with 10^5^ PFU of rZH501-M847-G, and livers, spleens and brains were isolated every day through day 8 p.i. To eliminate the possible contamination by rZH501-M847-A in the samples, rZH501-M847-A was not used in the BSL-4 laboratory during the experiments. For each sample, the virus titers, production of RT-PCR products and susceptibilities of the RT-PCR products to Sac I digestion are summarized in Fig. 8A and the representative data concerning the Sac I digestion of the RT-PCR products are shown in Fig. 8B. Most samples carrying detectable levels of infectious viruses and many samples in which virus titers were below the detection limit yielded the RT-PCR products. Accumulation of rZH501-M847-A-type virus was detected in the liver of a single mouse as early as 2 days p.i. and rZH501-M847-A-type virus became the major virus population in livers, spleens and brains from day 5 to 8 p.i. We performed sequence analysis of some of the cloned RT-PCR products to firmly establish the accumulation of rZH501-M847-A-type virus in rZH501-M847-G-infected mice; the numbers of the clones used for sequence analysis and the samples were: 20 clones from mouse 8b brain, 10 clones from mouse 6c spleen, 10 clones from mouse 7c brain, and 10 clones from mouse 7c spleen (see Fig. 8A). Consistent with the data of Sac I digestion of the RT-PCR products of these samples, all clones had A at M847. These data unambiguously demonstrated that rZH501-M847-A-type virus became the major virus population in rZH501-M847-G-infected mice.

To know whether the phenomenon of emergence of a major population of rZH501-M847-A-type virus after infection with rZH501-M847-G-type virus also occurred in cell culture, rZH501-M847-G was independently passaged five times at a multiplicity of infection (moi) of 0.01 and 1 in both VeroE6 cells and MRC-5 cells. RT-PCR products, including M847, were generated from intracellular RNAs, extracted from cells infected with viruses at passage levels 1 and 5, and subjected to Sac I digestion. We did not observe the accumulation of rZH501-M847A-type virus after 5 passages of rZH501-M847-G in both cell types (data not shown), demonstrating that the rZH501-M847-A-type virus did not emerge after passages of rZH501-M847-G in cultured cells but it became the major virus population in mice that were infected with rZH501-M847-G.

**Figure 9:**
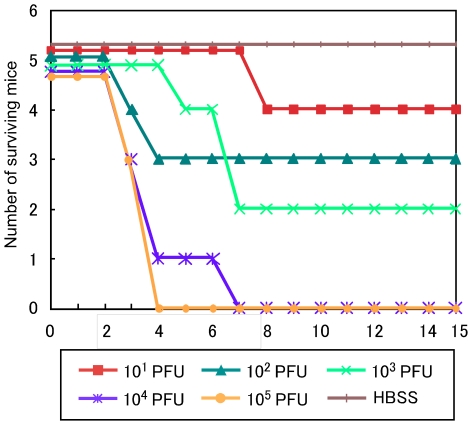
The virulence of rZH501-M847-A-type virus obtained from the brain of rZH501-M847-G mouse. Five 5-week-old female CD-1 mice were inoculated i.p. with 10^1^, 10^2^, 10^3^, 10^4^ or 10^5^ PFU of the brain homogenates from the 8b mouse (Fig. 8A). Control mice were inoculated with HBSS. Mortality was recorded daily for 28 days p.i. No mice died after 9 days p.i.

Finally, we examined mouse virulence of rZH501-M847-A-type virus that had accumulated in rZH501-M847-G-infected mice by i.p. inoculation of 10^1^, 10^2^, 10^3^, 10^4^ or 10^5^ PFU of the brain homogenates of 8b mouse (see Fig. 8A) into mice (n = 5 for each dilution); RT-PCR analysis of mouse 8b brain extracts revealed that rZH501-M847-A-type virus was the major virus population, and sequence analysis of all 20 cloned RT-PCR products of this brain extract showed that M847 was A. Inoculation of 10^4^ or 10^5^ PFU of the recovered virus resulted in the death of all mice within 7 days p.i., while 2 mice, 3 mice and 4 mice survived after inoculation of 10^3^ PFU, 10^2^ PFU and 10^1^ PFU of the virus, respectively (Fig. 9). The recovered viruses had a higher mouse virulence than did rZH501-M847-G, while they were not as virulent as rZH501-M847-A or ZH501 (Fig. 3).

## Discussion

A past report using reassortant viruses between an attenuated MP-12 strain and wt RVFV suggested that RVFV virulence in mouse is under polygenic control [13]. Past studies revealed that the NSs gene, encoded in the S segment, and the NSm gene, encoded in the M segment, both contribute to viral virulence [16], [17], [18]. We presented here our findings that a single nucleotide difference in M847 of the Gn gene of ZH501 substantially affected virulence in mice, a result that further supports the notion that RVFV virulence is under polygenic control [13].

Roughly a 1-to-1 mixture of the rZH501-M847-G-type virus and rZH501-M847-A-type virus existed in the ZH501 virus stock. Because the original ZH501 sample from a patient in the 1977 RVFV outbreak was not available, it is unclear whether high titers of both rZH501-M847-G-type virus and rZH501-M847-A-type virus replicated in the patient. We found that the rZH501-M847-A-type virus became a major viral population in mice inoculated with rZH501-M847-G, while viruses accumulated in mice inoculated with rZH501-M847-A were the rZH501-M847-A-type virus. Additionally, 38 of 39 isolates from wt RVFV thus far reported were found to have A at M847 and carry Glu277 [19], which is also encoded by rZH501-M847-A. These data suggest that the rZH501-M847-A-type virus probably represented the major virus population in the patient. However, it is possible that rZH501-M847-G-type virus existed as an RVFV quasispecies in the patient and affected the severity of the disease; quasispecies have cooperative interactions to control viral pathogenesis [20]. Consistent with this notion, we found a lesser virulence in a virus sample consisting of a major population of rZH501-M847-A-type virus and a minor population of rZH501-M847-G (Fig. 8A) and obtained from a rZH501-M847-G-infected mouse brain at 8 days p.i., than did rZH501-M847-A or ZH501 (Figs. 3 and 9). Although the accumulation of rZH501-M847A-type virus did not occur even after 5 passages of rZH501-M847-G in VeroE6 cells and MRC-5 cells, we observed that rZH501-M847-G replicated to higher titers than rZH501-M847-A in cultured cells (Figs. 2 and S1). This trend in virus replication in vitro pointed to the possibility that the rZH501-M847-G-type virus that was present as a minor virus population in the patient could have amplified in cell culture to become a major viral subpopulation in the ZH501 virus stock that was generated in vitro.

The kinetics of virus accumulation in various organs in mice inoculated with ZH501, rZH501-M847-A or rZH501-M847-G showed that RVFV replicates in the liver early in infection. The efficient replication of rZH501-M847-A and ZH501 in the liver and in other initial target organs probably induced high viremia titers that facilitated the spread of the virus to other organs, including the spleen, kidneys and brain ultimately leading to death of the animals. The virus titers in the liver of rZH501-M847-G-infected mice at 2 days p.i. were roughly ten times lower than those in the liver of rZH501-M847-A-infected mice (Fig. 4). Likewise, virus titers in other organs and serum of the rZH501-M847-G-infected mice were substantially lower than those of the corresponding organs of rZH501-M847-A-infected mice. The present data imply that the level of virus titers in the initial target organ, e.g., the liver, at 2 to 3 days p.i. may determine the severity and ultimate outcome of RVFV infection in mice.

Although we have demonstrated the importance of single nucleotide substitution at M847 of Gn protein for wt RVFV mouse virulence, further studies are required to determine how this single nucleotide substitution affected RVFV's virulence. Past studies showed that the gain-of-positive-charge mutations in the flavivirus E protein [21], [22], [23] and the alphavirus E2 protein [24] can enhance binding to negatively charged glycosaminoglycans (GAG) and increase the efficiency of virus uptake into cultured cells. However, the virulence of the variant viruses in mice is reduced, as these variant viruses are rapidly cleared from the circulatory system [22], [24], [25], [26]. Probably, the gain-of-positive-charge mutations in the envelope protein facilitates viral adherence to GAGs present in blood cells and subsequent reticuloendothelial system-mediated virus elimination from the circulation. Likewise, rZH501-M847-A, which had a negatively charged Glu277 of the Gn envelope protein, was highly virulent for mice, while rZH501-M847-G carrying an uncharged Gly277 was substantially less virulent (Fig. 3). Furthermore, rZH501-M847-A-infected mice showed higher viremia titers than rZH501-M847-G-infected mice at 1-3 days p.i. (Fig. 4), but rZH501-M847-G replicated at a higher titer than did rZH501-M847-A in MRC-5 cells (Fig. 2). Accordingly, it is possible that rZH501-M847-G binds efficiently to GAGs in the circulation, resulting in low levels of systemic infection, whereas rZH501-M847-A carrying Glu277 binds to GAGs less efficiently, resulting in high titers of viremia, efficiently establishes a systemic infection that involves the CNS, and kills the infected mice. The attenuated virus, MP-12, developed by serial passage of the wt RVFV ZH548 strain in the presence of 5-fluorouracil [27], carries A at M847 and encodes Glu277, suggesting that the presence of Glu277 in Gn does not always make RVFV virulent. The amino acid sequence of the M segment of MP-12 differed from that of rZH501-M847-A by 5 amino acids. We suspect that a combination of some or all of these mutated amino acids in the M segment contributed to the attenuation of MP-12.

The present study showed the importance of the A nucleotide at M847 of wt RVFV in mouse virulence, whereas it is unclear whether this nucleotide also affects viral virulence in other animals. Like rZH501-M847-G, one wt RVFV isolate 763/70, which was obtained from an aborted bovine fetus in Zimbabwe in 1970 [28], has G at M847 and carries Gly 277 [19], while all the rest of the wt RVFV isolates thus far sequenced carry A at M847 and encode Glu277 [19]. No information is available as to the titers of 763/70 in different tissues of the aborted fetus and the viremia status of its mother. Although we reported that pregnant cows inoculated with ZH501 become febrile, have 2.3 to 4.0 log_10_ PFU/ml of viremia, and undergo abortion [29], the relationship between level of viremia and abortion in cattle has not been carefully examined yet. We observed that very low viremia can result in abortion in experimentally infected sheep with ZH501 (J. Morrill, unpublished data), and, hence, we speculate that placental tissue in sheep and probably cattle may be exquisitely sensitive to infection, and low titers of viremia can infect the placenta at very low levels and gain access to the fetus. Virulence of 763/70 in the bovine has not been experimentally tested and 763/70 differs from ZH501 at other genome regions. Accordingly, the role of G at M847 of 763/70 in virus virulence for bovine is unclear. Experimental infection of rZH501-M847-A and rZH501-M847-G into domestic animals will show whether the importance of the A nucleotide at M847 in viral virulence is limited to the mouse model.

A remarkably rapid emergence and accumulation of rZH501-M847-A-type virus occurred in the mice inoculated with rZH501-M847-G (Figs. 7 and 8). In the past, an experimental infection using swine influenza virus resulted in a similarly rapid emergence of a variant virus in infected hosts during single infections [30]. There are other examples of this type of quasispecies emergence and fixation in vivo [31], [32], [33]. Although rZH501-M847-G was rescued from cDNAs and direct sequencing of the M segment of rZH501-M847-G did not show the presence of rZH501-M847-A-type in the inoculum (data not shown), rZH501-M847-A-type viruses might have been generated during T7 polymerase-mediated primary transcription from the plasmid or during M segment RNA replication and existed as one of a minor virus population of ZH501-M847-G quasispecies in the inoculum. Alternatively, rZH501-M847-A-type virus was generated by a point mutation at M847 of the M segment in the rZH501-M847-G-infected mice and became a major virus population as early as 2 days p.i. As discussed above, we speculate that rZH501-M847-A may bind to GAGs less efficiently than does rZH501-M847-G. This putative phenotype of rZH501-M847-A-type virus might have prevented the efficient elimination of the virus from reticuloendothelial systems, and facilitated continuous replication and systemic infection.

We noted that neutralizing antibodies were rapidly elicited in rZH501-M847-G-infected mice within 3 days p.i. (Fig. 5), during which time most of the viruses retained the genotype of rZH501-M847-G (Fig. 8). Because the importance of neutralizing antibodies for the protection of animals from RVFV infection is well documented [34], [35], [36], [37], [38], [39], [40], [41], [42], a rapid and efficient generation of neutralizing antibodies probably contributed to low titers of viremia and prevention of systemic infection in rZH501-M847-G-infected mice. It is interesting to note that the antiserum neutralized the heterologous virus equally but to a lesser degree than the homologous virus in the cross neutralization assay (Table 1). These results were consistent with a report that the region including Glu277 or Gly277 of Gn protein is a major neutralizing epitope site [43]. It is likely that in the rZH501-M847-G-infected mice, rZH501-M847-A-type virus emerged partly because this virus was less susceptible to rapidly generated neutralizing antibodies directed against rZH501-M847-G. We further suspect that in the rZH501-M847-G-infected mice, newly emerged rZH501-M847-A-type virus failed to continuously replicate efficiently after 5 days p.i. in various organs primarily due to an increase in the titers of neutralizing antibodies, resulting in the survival of the mice. The virus titers in rZH501-M847-A-inoculated mice were higher than those inoculated with rZH501-M847-G in many organs during the first 4-5 days p.i., while the neutralizing antibody titers tend to be higher in rZH501-M847-G-inoculated mice than in rZH501-M847-A-inoculated mice (Fig. 5). These data suggest that rZH501-M847-A replication might have induced immunosuppression. This possibility was consistent with the data that lymphocytes were depleted in the white pulp of mice inoculated with rZH501-M847-A, but not those inoculated with rZH501-M847-G, a finding probably due to apoptosis (Fig. 6).

## Materials and Methods

### Viruses and cells

The ZH501 strain of wt RVFV was obtained from the Special Pathogens Branch at the Centers for Disease Control and Prevention (CDC) (Atlanta, Georgia). RVFV ZH501 was originally isolated from a patient during the 1977 outbreak of RVFV in Egypt by inoculating the human specimen into suckling mice brain. After amplification of the virus in the suckling mice brain one more time, the ZH501 virus stock was generated after two passages in FRhL cells and two passages in VeroE6 cells. The Vero cell line and MRC-5 cell line were used for a viral plaque assay as described previously [44]. All of the experiments that used virulent ZH501 were performed in The Robert E. Shope, MD BSL-4 Laboratory in the John Sealy Pavilion for Infectious Diseases Research at The University of Texas Medical Branch at Galveston, Texas.

### Plasmid constructions

Standard molecular biological techniques were used to construct plasmids. Total RNA of VeroE6 cells infected with RVFV ZH501, which was obtained from Stuart Nichol at the CDC, was used for first-strand cDNA synthesis. ZH501 virus stock at CDC [16] and our ZH501 stock underwent the same passage history. PCR fragments of full-length, antiviral-sense ZH501 S and L-segments were cloned into a pProT7 plasmid [45], designated as pProT7-wS(+) and pProT7-wL(+), respectively. The ZH501 M-segment carrying G and A at nucleotide 847 of anti-viral-sense was cloned into a pProT7 plasmid, designated as pProT7-wM(+)-M847-G and pProT7-wM(+)-M847-A, respectively. The plasmids expressing ZH501 N or L proteins under the control of T7 RNA polymerase were reported previously [46]. The ORF of ZH501 glycoproteins, which carry G at nucleotide 847 of anti-viral-sense M were cloned into the pCAGGS plasmid under chicken β-actin promoter, designated as pCAGGS-G. All of the constructs were confirmed to have the expected sequences.

### Virus rescue

rZH501-M847-G, a recombinant ZH501 carrying G at nucleotide 847 of anti-viral-sense M, was recovered by co-transfection of pProT7-wS(+), pProT7-wM(+)-M847-G, pProT7-wL(+), pT7-IRES-N, pT7-IRES-L and pCAGGS-G into BHK/T7-9 cells by TransIT-LT1 (Mirus, Madison, WI), as described previously [45]. rZH501-M847-A was recovered by co-transfection of pProT7-wS(+), pProT7-wM(+)-M847-A, pProT7-wL(+), pT7-IRES-N, and pT7-IRES-L into BHK/T7-9 cells. The culture medium was replaced with fresh medium at 24 h post transfection. At 5 days post transfection, the culture supernatants were collected, clarified and then inoculated into VeroE6 cells. The supernatant of infected VeroE6 cells at 3 days p.i. was collected and used for titration of virus infectivity by plaque assay and for the subsequent experiments. Sequence analysis of M segment RNA confirmed the presence of the expected nucleotide at M847 in rZH501-M847-G and rZH501-M847-A.

### Sequence analysis of the rescued viruses

Intracellular viral RNAs of infected MRC-5 cells served as the source of sequence analysis. Most of the sequences of the L, M and S RNA segments of rescued rZH501-M847-A and rZH501-M847-A were determined by direct sequencing analysis of the virus-specific RT-PCR products. For sequencing of the 5′-end of viral RNAs, free 5′-phosphates in intracellular RNAs were removed by calf intestinal alkaline phosphatase and then the samples were treated with tobacco acid pyrophosphatase to remove the cap structure. After ligation of the RNA adaptor to uncapped RNA by using T4 RNA ligase, cDNA was synthesized by employing an RLM-RACE kit (Ambion, Austin, TX) using random primers. Amplicons were obtained by using a virus-specific and an adaptor-specific primer and subjected to sequencing analysis. For sequencing of the 3′-end of viral RNAs, a poly A tail was added to viral RNAs by using a Poly(A) Polymerase Tailing Kit (Epicenter biotechnology, Madison, WI). First-strand cDNA was synthesized from RNA containing a poly A tail by using the 3′Race adaptor (RLM-RACE kit, Ambion). The cDNA was subjected to PCR with an adaptor-specific primer and a virus-specific primer, and the amplicons were subjected to sequence analysis.

### Analysis of viral growth

VeroE6 cells, mouse 3T3 cells, mouse macrophage-derived J774.1 cells and MRC-5 cells were infected with viruses at an moi of 0.02 at 37°C for 1 h, and washed 3 times with PBS. Then medium was added. Culture supernatants were harvested at various times p.i., and the virus titer was measured by plaque assay.

### Virus plaque assays

Virus titers were determined by plaque assay in Vero cell monolayers grown in 24-well plates. For the viral plaque assay, serial tenfold dilutions of each specimen were prepared in HBSS with 2% fetal bovine serum added (HBSS-FBS), and 50 µl of each dilution was adsorbed on duplicate cell monolayers for 1 hour at 37°C. After 1 hour, the monolayers were overlaid with 0.6 ml of a mixture of 1 part 1% agarose and 1 part 2X Eagle's basal medium with Earle's salts, 17 mm HEPES, 8% fetal bovine serum, 100 U of penicillin/ml and 100 µg of streptomycin sulfate/ml. After incubation for 72 h at 37°C in a 5% CO_2_ atmosphere, each cell monolayer was stained by adding 0.6 ml of a second overlay, identical to the first, but containing 4% neutral red. After an additional 24 h incubation, plaques were enumerated and viral titers were calculated.

### Virus inoculation into mice and collection of samples from infected mice

Five-week-old female outbred CD-1 mice were purchased from Charles River Laboratories (Wilmington, MA). The mice were housed, 5 mice per cage, in micro-isolator cages, in a BSL-4 biological containment laboratory with a 12-h day-night photoperiod. Virus inocula were prepared in HBSS, pH 7.4, with no supplements. Each mouse was inoculated i.p. with 0.1 ml of inoculum in a tuberculin syringe fitted with a 26-gauge, 3/8- inch needle. Moribund mice or mice pre-selected for euthanasia were anesthetized with isoflurane, whole blood was collected via cardiac puncture, and the mice were euthanatized by cervical dislocation. Liver, spleen, and brain tissues were aseptically collected immediately following euthanasia or as soon as dead mice were discovered. All experiments were performed in accordance with guidelines of the Institutional Animal Care and Use Committee of the University of Texas Medical Branch and the recommendations in the *Guide for the Care and Use of Laboratory Animals* (Institute of Laboratory Animal Resources, National Research Council, National Academy of Sciences, 1996). The facilities used are fully accredited by the American Association for Accreditation of Laboratory Animal Care.

### Preparation of organ homogenates and sera

Tissues were prepared as 10% (w/v) homogenates in HBSS supplemented with 10% heat-inactivated (56°C for 30 minutes) fetal bovine serum and antibiotics (200 U/ml penicillin and 50 µg/ml streptomycin). Serum for virus and neutralizing antibody assays was separated from whole blood by centrifugation at 1,500 x g for 15 minutes and stored at -80°C until assayed. Tissue homogenates were similarly clarified by centrifugation, and supernatants were harvested and stored at −80°C until assayed.

### Neutralizing antibody assays

Serum neutralizing antibody was determined using an 80% plaque-reduction neutralization test. Serial fourfold dilutions of serum were prepared in HBSS-FBS. An equal volume of the ZH501 strain of RVFV suspension containing approximately 80 PFU/50 µl was added to each dilution. After incubation at 37°C for 1 hr, 50 µl of each dilution was adsorbed on duplicate Vero cell monolayers for 1 hr at 37°C and then overlaid with 0.6 ml of the agarose-medium mixture used in the viral plaque assay. After 72 hr incubation at 37°C in a 5% CO_2_ atmosphere, each monolayer received 0.6 ml of a second agarose containing neutral red dye. Plaques were counted and an 80% reduction in the number of plaques inoculated was used as the endpoint for virus-neutralization titers.

### Cross-neutralization assays

Anti-rZH501-M847-G serum demonstrating a PRNT_80_ titer of 1∶640 to ZH501 was a mixture of the sera, each collected at days 6, 7 and 8 p.i. from eight rZH501-M847-G-infected mice. Similarly, anti-rZH501-M847-A serum demonstrating a PRNT_80_ titer of 1∶80 to ZH501 was a mixture of the sera collected at 6 days p.i. from four rZH501-M847-A-infected mice. Diluent (HBSS-FBS) and normal mouse serum served as negative controls, while convalescent goat anti-ZH501 serum showing a PRNT_80_ titer of 1∶5,120 to ZH501 served as a positive control. We incubated at 4°C overnight vials containing 100 µl of approximately 5.0 log_10_ PFU/ml of rZH501-M847-G, rZH501-M847-A or ZH501 combined with 100 µl of each serum sample or diluents. After incubation, virus titers were determined by using viral plaque assays.

### RNA extraction from organs

One hundred microliters of 10% tissue homogenate were mixed with 900 µl of TRIzol reagent (Invitrogen, Carlsbad, CA). After addition of 200 µl of chloroform, tubes were shaken vigorously by hand and centrifuged at 15,000 rpm for 10 min at 4°C. Following centrifugation, the aqueous phase was transferred to a new tube and 500 µl of isopropanol was added to the tubes. Samples were centrifuged at 15,000 rpm for 25 min at 4°C. RNA pellets were washed with 75% ethanol and dried. Thirty microliters of RNase-free water was added to dissolve the RNA pellet. The samples were then treated with RQ1 RNase-Free DNase (Promega, Madison, WI), and the RNAs were purified by addition with phenol-chloroform.

### RT-PCR and Sac I digestion

The total RNA of infected VeroE6 cells or mouse liver, spleen, kidneys and brain were extracted with Trizol reagent (Invitrogen). First-stranded cDNA was synthesized with a random hexamer by RTG YouPrime RXN Beads (GE Healthcare, Bucks, UK) according to the manufacturer's instructions. PCR primers which anneal to nucleotide 411 to nucleotide 430 (M430F: 5′-ATG GCA GGG ATT GCA ATG AC-3′) or nt.1041 to 1060 (M1041R: 5′-ACT GCA AAG GGC ACA ACC TC-3′) of anti-viral-sense M were used for PCR reaction. PCR was performed for 30 cycles at 94°C for 40 sec, 55°C for 1 min, and 72°C for 1 min using the Expand High Fidelity PCR System (Roche, Mannheim, Germany). The PCR products were purified with QIAquick PCR Purification Kit (Qiagen, Germantown, MD), digested with Sac I and then separated on a 1% agarose gel.

### Sequence of ZH501 M-segment

The PCR product consisting of a wild-type ZH501 M-segment by M430F and M1041R was directly sequenced, or cloned into pSTBlue-1 by AccepTor Vector Kits (Novagen, Darmstadt, Germany) according to the manufacturer's instruction. Thirty-five clones were sequenced by T7 primer.

### Histopathology and IHC examination

Specimens for histopathologic examination were collected in 10% neutral buffered formalin. The livers, spleens, kidneys, and brains obtained from infected mice and control animals were processed for histopathological and IHC examination as previously described [47]. Formalin-fixed and paraffin-embedded tissue sections were subjected to hematoxylin and eosin (H&E) by standard methods for evaluating histopathology and IHC staining for detecting RVFV antigens, respectively. For detecting RVFV antigens, the tissues were incubated with rabbit anti-N antibody [48] (1∶500). Color was developed by using the fuchsin+ substrate-chromogen system (DAKO cytomation, Carpentaria, CA).

## Supporting Information

Figure S1 Growth curve of rZH501-847-A and rZH501-847-A in MRC-5 cells. MRC-5 cells were inoculated with rZH501-847-A or rZH501-847-A at an moi of 0.02. Culture fluids were collected and virus titers were determined by a plaque assay that used VeroE6 cells. The results were obtained from three independent experiments.
(0.07 MB TIF)

Figure S2 Histopathology and IHC of mice infected with rZH501-M847-A (A to D), and rZH501-M847-G (E to H). (A) Brain of mouse infected with rZH501-M847-A on day 6 p.i. Viral antigens were detected by IHC in the neurons (magnification, ×200, inset: ×400). (B) Glomerulus of the kidney in mouse infected with rZH501-M847-A; some mice showed pyknotic cells (arrow) in glomeruli (magnification, ×600). (C, D) RVFV antigens were detected by IHC in the glomeruli (C) and blood smooth muscle of interlobular and arcuate arteries (D) of mice infected with rZH501-M847-A (magnification, ×600). (E) Mice infected with rZH501-M847-G had encephalitis on day 8 p.i. Viral antigens were detected in the neurons by IHC (magnification, ×200, inset: ×400). (F) No lesions were found in the kidneys of mice infected with rZH501-M847-G (magnification, ×600). (G, H) Viral antigens were detected in the smooth muscles of interlobular and arcuate arteries (H); however antigens were not detected in the glomeruli of mice infected with rZH501-M847-G (G) (magnification, ×600).
(2.55 MB TIF)
