# Correction: Rapid Accumulation of Virulent Rift Valley Fever Virus in Mice from an Attenuated Virus Carrying a Single Nucleotide Substitution in the M RNA

**DOI:** 10.1371/annotation/326c5f5b-3da9-4b0b-b889-28d4f33d2543

**Published:** 2010-04-13

**Authors:** John C. Morrill, Tetsuro Ikegami, Naoko Yoshikawa-Iwata, Nandadeva Lokugamage, Sungyong Won, Kaori Terasaki, Aya Zamoto-Niikura, C. J. Peters, Shinji Makino

The following information was missing from the Funding section. This study was supported by NIH/NIAID grant 1UO1-AI062636.

