# Supplementary figures and images for: Rapid Accumulation of Virulent Rift Valley Fever Virus in Mice from an Attenuated Virus Carrying a Single Nucleotide Substitution in the M RNA
Source: PLoS One. 2010 Apr 1;5(4):e9986. doi: 10.1371/journal.pone.0009986 (PMC2848673; doi:10.1371/journal.pone.0009986)

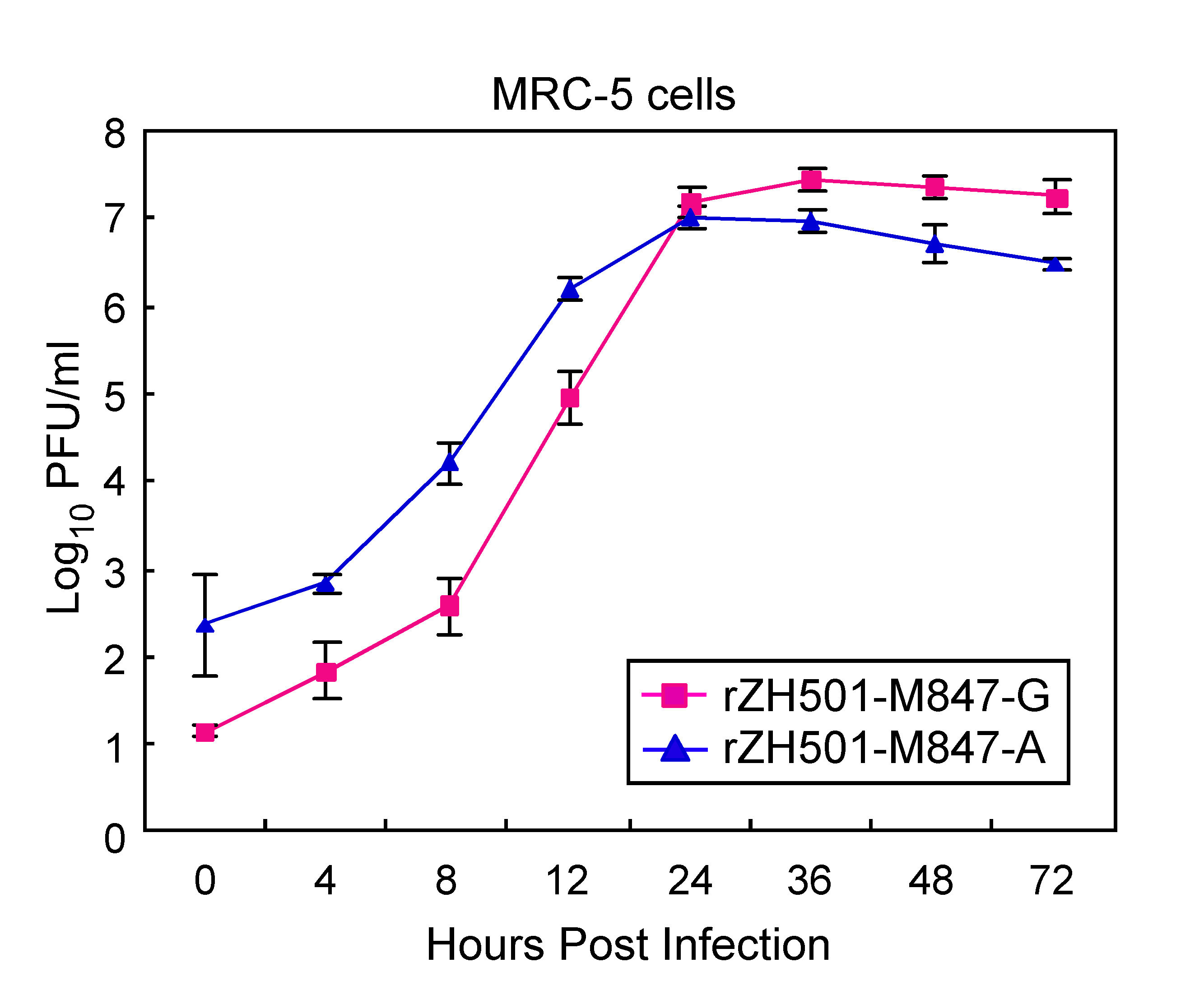

Supplement: Figure S1 — Growth curve of rZH501-847-A and rZH501-847-A in MRC-5 cells. MRC-5 cells were inoculated with rZH501-847-A or rZH501-847-A at an moi of 0.02. Culture fluids were collected and virus titers were determined by a plaque assay that used VeroE6 cells. The results were obtained from three independent experiments. (0.07 MB TIF) [file pone.0009986.s001.tif]

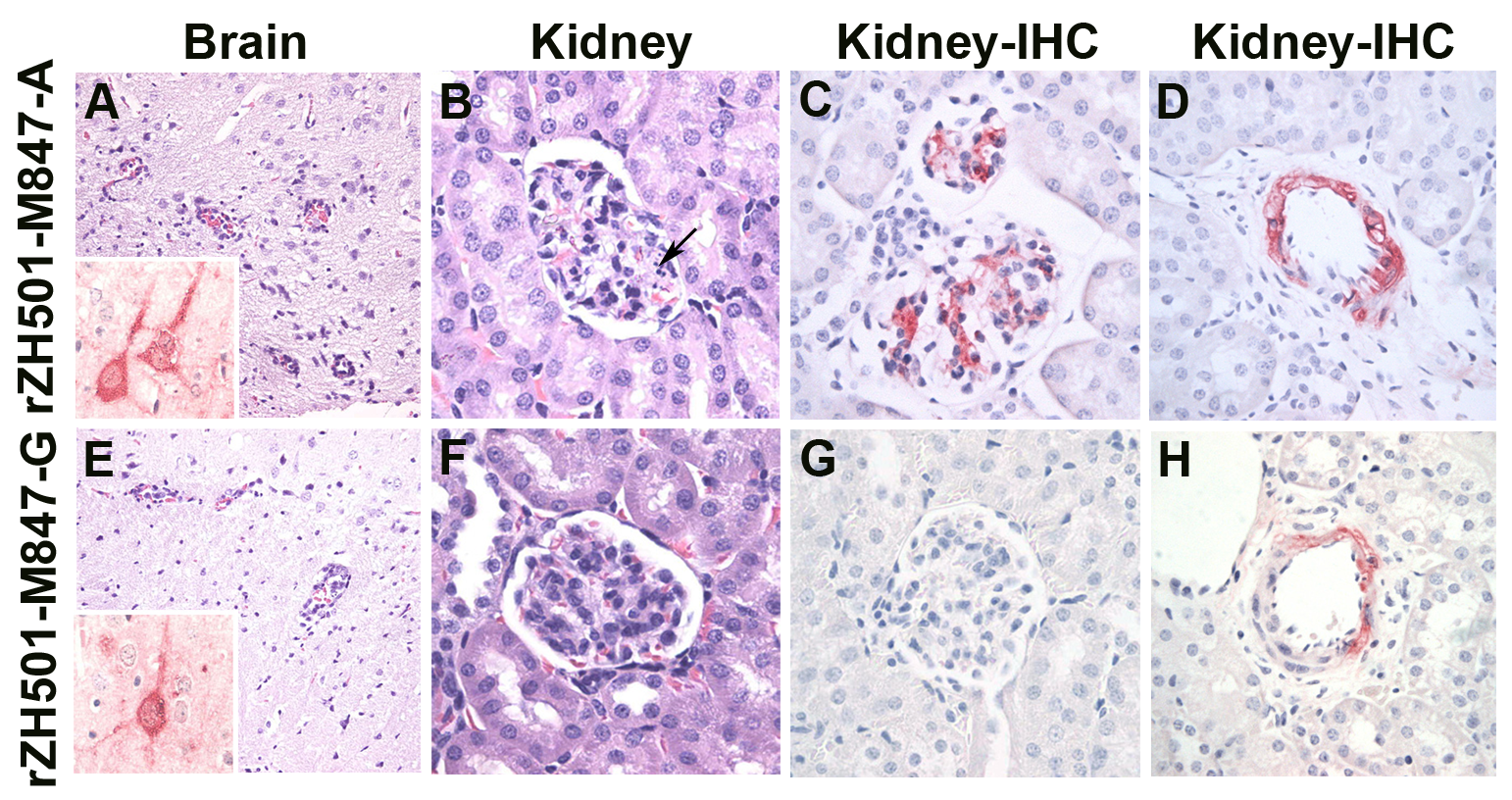

Supplement: Figure S2 — Histopathology and IHC of mice infected with rZH501-M847-A (A to D), and rZH501-M847-G (E to H). (A) Brain of mouse infected with rZH501-M847-A on day 6 p.i. Viral antigens were detected by IHC in the neurons (magnification, ×200, inset: ×400). (B) Glomerulus of the kidney in mouse infected with rZH501-M847-A; some mice showed pyknotic cells (arrow) in glomeruli (magnification, ×600). (C, D) RVFV antigens were detected by IHC in the glomeruli (C) and blood smooth muscle of interlobular and arcuate arteries (D) of mice infected with rZH501-M847-A (magnification, ×600). (E) Mice infected with rZH501-M847-G had encephalitis on day 8 p.i. Viral antigens were detected in the neurons by IHC (magnification, ×200, inset: ×400). (F) No lesions were found in the kidneys of mice infected with rZH501-M847-G (magnification, ×600). (G, H) Viral antigens were detected in the smooth muscles of interlobular and arcuate arteries (H); however antigens were not detected in the glomeruli of mice infected with rZH501-M847-G (G) (magnification, ×600). (2.55 MB TIF) [file pone.0009986.s002.tif]
